# ATTITUDES TOWARD AGING AND WELLNESS ENGAGEMENT IN LIFE PLAN COMMUNITIES

**DOI:** 10.1093/geroni/igac059.2011

**Published:** 2022-12-20

**Authors:** Jennifer Smith, Matthew Fullen, Philip Clarke

**Affiliations:** Mather Institute, Evanston, Illinois, United States; Virginia Tech, Blacksburg, Virginia, United States; Wake Forest University, Winston-Salem, North Carolina, United States

## Abstract

Life Plan Communities, also known as Continuing Care Retirement Communities, typically offer a wide range of wellness programs and services, including fitness classes, educational lectures, volunteer opportunities, and social events. Despite the convenience of onsite wellness resources, some residents choose not to participate in wellness offerings available at the community. One factor that may influence engagement in wellness behaviors is older adults’ attitudes toward aging, which has been associated with differences in health and well-being over time. The purpose of this research was to examine the relationship between attitudes toward aging and wellness engagement among residents of Life Plan Communities. A total of 447 residents (ages 59 to 97; M = 81.82, SD = 6.55; 65% female) of 10 communities completed surveys that measured their attitudes toward aging, self-reported wellness, interest in improving wellness, participation in wellness programs, and barriers to wellness participation. Multiple regression analyses revealed that more positive attitudes toward aging were associated with better wellness, higher interest, more frequent participation, and fewer barriers, controlling for age, gender health, education, and marital status. Follow-up analyses revealed that these effects could be accounted for by specific types of aging attitudes (i.e., psychological growth, psychosocial loss, and physical change). For example, greater psychological growth was associated with greater wellness, interest in improving wellness, and wellness participation, whereas greater psychosocial losses was associated with greater perceived barriers and lower wellness. These findings have implications for the development, implementation, and promotion of wellness programs for older adults.

